# Transcriptome Analysis Reveals Sugar and Hormone Signaling Pathways Mediating Flower Induction in Pitaya (*Hylocereus polyrhizus*)

**DOI:** 10.3390/ijms26031250

**Published:** 2025-01-31

**Authors:** Kamran Shah, Xiaoyue Zhu, Tiantian Zhang, Jiayi Chen, Jiaxuan Chen, Yonghua Qin

**Affiliations:** 1 Guangdong Provincial Key Laboratory of Postharvest Science of Fruits and Vegetables, College of Horticulture, South China Agricultural University, Guangzhou 510642, China; kamranshah801@scau.edu.cn (K.S.); 20223137243@stu.scau.edu.cn (X.Z.); chenjiayi98@stu.scau.edu.cn (J.C.); jxchen0127@163.com (J.C.); 2 Key Laboratory of Biology and Genetic Improvement of Horticultural Crops, Ministry of Agriculture and Rural Affairs, College of Horticulture, South China Agricultural University, Guangzhou 510642, China; 3 Department of Ecology, College of Natural Resources and Environment, South China Agricultural University, Guangzhou 510642, China; 17863608557@163.com

**Keywords:** auxin, flowering, gibberellin, hormone, pitaya, sugar

## Abstract

Flower induction in pitaya (*Hylocereus polyrhizus*) is regulated by complex gene networks involving multiple signaling pathways that ensure flower bud (FB) formation, but its molecular determinants remain largely unknown. In this study, we aimed to identify key genes and pathways involved in pitaya flower induction by analyzing transcriptomics profiles from differentiating buds. Our results indicate that the flower induction process is driven by a combination of sugar, hormone, transcription factor (TF), and flowering-related genes. We found that during the FB induction period, the levels of sugar, starch, auxin (AUX), cytokinin (CTK) active forms dihydrozeatin riboside (dhZR), zeatin riboside (ZR), N6-isopentenyladenosine (iPA), and brassinosteroid (BR) increase in the late stage (LS), while active gibberellins (GA3, GA4) decrease, signaling a metabolic and hormonal shift essential for flowering. Differential gene expression analysis identified key genes involved in starch and sugar metabolism, AUX, CTK, BR synthesis, and (GA) degradation, with notable differential expression in photoperiod (*COL*, *CDF*, *TCP*), age-related (*SPL*), and key flowering pathways (*FT*, *FTIP*, *AGL*, *SOC1*). This study reveals a multidimensional regulatory network for FB formation in pitaya, primarily mediated by the crosstalk between sugar and hormone signaling pathways, providing new insights into the molecular mechanism of FB formation in pitaya.

## 1. Introduction

Flower bud formation is a highly complex biological process that requires a pre-coordination of environmental and endogenous signals to ensure successful reproduction. In model plants like *Arabidopsis thaliana*, flowering is regulated through a variety of pathways, including the autonomous, vernalization, thermosensory, photoperiod, aging and gibberellic acid (GA) pathways [1]. This phase transition is pivotal for reproductive success as it marks the shift from vegetative to reproductive development, ultimately influencing fruit production [2,3]. However, the molecular mechanisms that regulate FB induction in perennial cactus species, such as *Hylocereus polyrhizus* (pitaya), differ considerably from those in Arabidopsis. For example, in Arabidopsis, GA promotes the transition to reproductive development [4], but in other species like *Citrus unshiu* [5], *Mangifera indica* [6], *Prunus avium* [7], and *Malus domestica* [8], GA inhibits flowering. Understanding the molecular determinants of FB induction in cactus plants is crucial for optimizing their cultivation and breeding.

Sugars are critical to the flowering process [9], not only providing energy for FB formation but also acting as signaling molecules. In species such as pitaya [10], *Vitis vinifera* [11], *Malus domestica* [12,13] and citrus [14], sugars like glucose, sucrose, and trehalose-6-phosphate regulate flowering [15] by being transported from the source leaves to the shoot apical meristem (SAM), where they play a role in FB induction [16]. In *Arabidopsis thaliana,* the TF *AtIDD8* influences sugar transport and metabolism, affecting the photoperiod pathway and flowering [17]. Additionally, the modulation of the *CONSTANS* (*CO*) gene through starch synthase genes, such as *GBSSI*, underlies another mechanism through which sugars contribute to flowering [18]. Despite these advances, the specific roles of sugar and sugar synthesis-related genes during the FB induction period in pitaya remain poorly understood.

Plant hormones also play an indispensable role in regulating flowering. In Arabidopsis, AUX activates the expression of *ARF4*, which, in turn, promotes key floral meristem identity genes like *APETALA1* (*AP1*) and *FRUITFULL*, facilitating the transition from vegetative to reproductive development [19]. Cytokinins, GAs, and BRs have also been identified as critical regulators. For instance, the constitutive expression of *MADS-box* genes results in early flowering via the CTK and GA signaling pathways [20]. *FLOWERING LOCUS T* (*FT*) and its homolog *TWIN SISTER OF FT* are essential in mediating the flowering response to CTK [21]. Furthermore, GAs not only promote flowering but also exert an inhibitory effect by modulating DELLA proteins, showcasing their dual role in the process [4]. However, the concentrations and specific role of key hormones during the FB induction period in pitaya remain largely unidentified.

*CONSTANS*, *APETALA2* (*AP2*), and *FT* are key regulators in olive (*Olea europaea*) flowering induction. *CONSTANS* likely mediates photoperiodic control of flowering through *FT* activation, essential for the transition to reproductive stages. *APETALA2* TF influences hormone-related pathways, modulating bud differentiation. Overexpression of *FT* and related genes drives floral differentiation, highlighting their roles in the vegetative-to-reproductive switch and offering potential targets for breeding programs to reduce alternate bearing and enhance consistent productivity [22].

Pitaya (*Hylocereus polyrhizus*) is a long-day, tropical fruit known for its rich nutritional profile, including betalains, antioxidants, vitamins, fiber, and minerals [23,24]. Despite its economic importance, there have been limited studies on pitaya flowering, with only four studies addressing light-induced flowering [10,25], the use of phytotrons at specific temperatures [26], and gene identification during bud growth stages [27]. However, these studies suffer from practical limitations, such as the lack of commercial-scale light supplementation and resource-intensive experimental setups. Thus, there is a clear gap in understanding the molecular determinants of flowering in pitaya.

Our study aims to fill this gap by identifying the FB formation period in *Hylocereus polyrhizus* in Guangzhou, China, which spans from 8 to 28 April. Through sampling at the start, middle, and end of this period, we explored the metabolic, hormonal, and genetic changes associated with the flowering induction phase. Our results provide new insights into the roles of sugars and hormones in the flowering process of pitaya, highlighting key molecular mechanisms involved in the transition to reproductive development.

## 2. Results

### 2.1. Dynamic Changes in Sugar Levels in Pitaya Bud During Flower Induction

**Figure 1:**
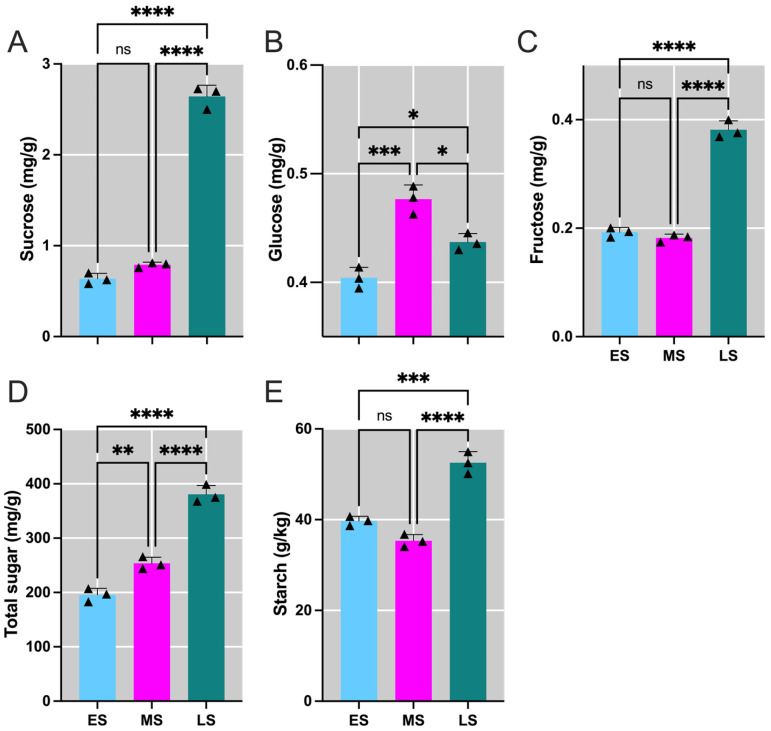
Temporal variations in the sugar levels in *Hylocereus polyrhizus* buds throughout flower bud induction period. Panels show the concentrations of (**A**) sucrose, (**B**) glucose, (**C**) fructose, (**D**) total sugar, and (**E**) starch. The stages of flower bud induction are represented by the early stage (ES), middle stage (MS), and late stage (LS). Data are presented as mean ± SD for three replicates (*n* = 3). Small triangles represent the distribution of data for each biological repeat. Significance levels are denoted as follows: * *p* < 0.05; ** *p* < 0.01; *** *p* < 0.001; **** *p* < 0.0001; nonsignificant differences are indicated by ‘ns’ (*p* > 0.05).

In *H. polyhrizus*, sugar levels such as sucrose, fructose, total sugar, and starch significantly increased in the LS of flower induction period compared to the ES and MS (Figure 1A,C–E). Glucose levels were significantly higher in the MS compared to the ES but remained comparable to those in the LS (Figure 1B). These changes indicate that sugars and starch play a crucial role in regulating the flowering process.

### 2.2. Dynamic Changes in Hormone Levels in Pitaya Bud During Floral Induction

**Figure 2:**
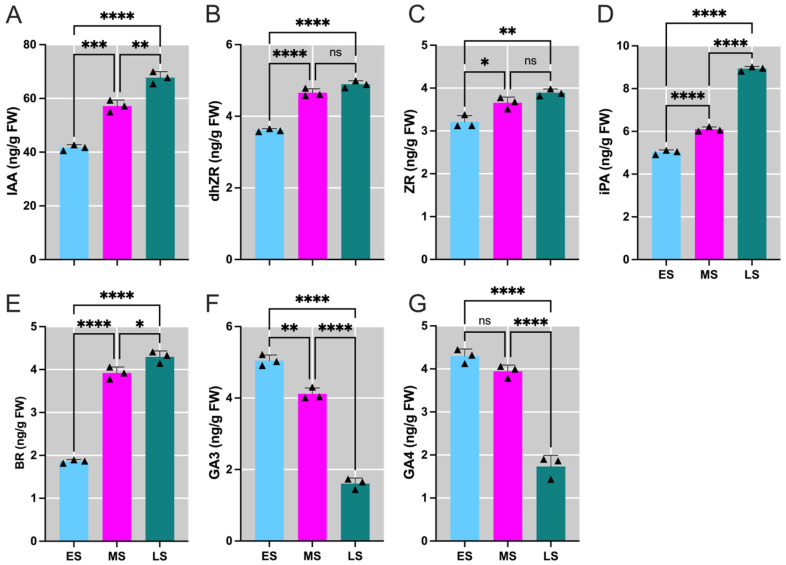
Temporal fluctuations in plant hormone levels in *Hylocereus polyrhizus* buds throughout the flower bud induction period. Panels show the concentrations of (**A**) Auxin (AUX), (**B**) Dihydrozeatin riboside (dhZR), (**C**) Zeatin riboside (ZR), (**D**) N6-isopentenyladenosine (iPA), (**E**) Brassinosteroid (BR), (**F**) Gibberellin-3 (GA3), and (**G**) Gibberellin-4 (GA4). The stages of flower bud induction are represented by the early stage (ES), middle stage (MS), and late stage (LS). Data are presented as mean ± SD for three replicates (*n* = 3). Small triangles represent the distribution of data for each biological repeat. Significance levels are denoted as follows: * *p* < 0.05; ** *p* < 0.01; *** *p* < 0.001; **** *p* < 0.0001; nonsignificant differences are indicated by ‘ns’ (*p* > 0.05).

Plant hormones play a crucial role in the transition to the reproductive phase. In pitaya, four key hormones were analyzed. The levels of AUX, active CTKs (dhZR, ZR, iPA), and BR increased from ES to LS (Figure 2A–E), while the levels of active GAs (GA3 and GA4) decreased (Figure 2F,G). These hormonal changes highlight their regulatory impact on pitaya flowering.

### 2.3. Analysis of Differentially Expressed Genes (DEGs)

**Figure 3:**
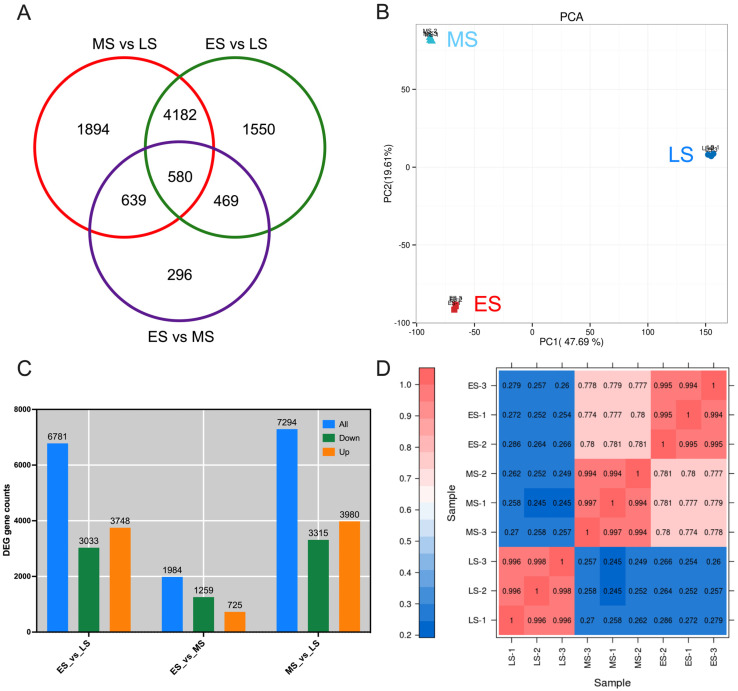
Multifaceted analyses of gene expression in *Hylocereus polyrhizus* buds throughout flower bud induction period. (**A**) Venn diagram illustrating the intersecting and unique differential expressed genes (DEGs) among the ES, MS and LS, referring to the early, middle, and late stages. (**B**) Principal component analysis plot delineating gene expression variability among the stages. (**C**) Distribution of DEGs across all groups, with the X-axis indicating comparison groups and the Y-axis showing gene counts. Blue bars represent total regulated genes, green bars indicate downregulated genes, and orange bars denote upregulated genes. (**D**) Relationship analyses of the samples. ES: early stage; MS: middle stage; LS: late stage. Numbers 1, 2, and 3 represent biological repeats for each stage: ES1, ES2, ES3 for early stage; MS1, MS2, MS3 for middle stage; and LS1, LS2, LS3 for late stage. ES: early stage; MS: middle stage; LS: late stage.

To uncover the molecular mechanisms behind variations in sugar, starch, and hormone levels during the transition to the reproductive phases, and to identify key gene expressions mediating these changes, buds from distinct stages of the FB induction period were subjected to transcriptome sequencing analysis. Buds representing the ES, MS, and LS of the flower induction period were sequenced, with three replicates per sample to ensure data reliability. Clean reads per library ranged from 31.88 to 36.96 million, with Q20 and Q30 percentages above 96% and 92%, respectively, and a GC content over 47% (Supplementary Table S3). Unique mapping rates to the pitaya genome varied from 13.37% to 64.46%, and the total reads, mapped reads, and multiple mapped read ratios were 63.76–73.93 million, 14.11–68.73%, and 0.74–4.26%, respectively. Positive and negative read maps were 7.53–37.04% and 7.56–37.22%, respectively (Supplementary Table S4). The Venn diagram (Figure 3A) showed 580 DEGs common to all stages. Unique DEGs included 1894 for the MS vs. LS, 1550 for the ES vs. LS, and 296 for the ES vs. MS. Shared DEGs were 639 for the ES-MS, 4182 for the MS-LS, and 469 for the ES-LS, indicating stage-specific and overlapping gene expressions. Principal component analysis (PCA) (Figure 3B) displayed distinct clustering of the ES, MS, and LS samples, indicating significant transcriptional diversity. The ES samples clustered in the negative quadrants of PC1 and PC2, the LS samples in the positive quadrants, and the MS samples near LS on PC1 but neutral on PC2, suggesting similarities between the MS and LS distinct from the ES. DEG analysis (Figure 3C) revealed 6781 DEGs between the ES and LS (mostly upregulated), 1984 DEGs between the ES and MS (mostly downregulated), and 7295 DEGs between the MS and LS (predominantly upregulated), indicating substantial transcriptional shifts. Pearson’s correlation analysis (Figure 3D) showed high reproducibility among biological replicates (R2 > 0.99). Low correlations were observed between the ES and LS and between the LS and MS (R < 0.29), while the ES and MS had a moderate correlation (R > 0.77), indicating some similarity in gene expression profiles.

KEGG analysis of DEGs showed significant involvement in plant hormone signal transduction and starch and sucrose metabolism. Among 1756 DEGs, 55.35% were upregulated and 44.65% were downregulated, highlighting these pathways as predominant (Table 1).

### 2.4. Alterations in Sugar Metabolism and Signaling Pathways in Pitaya Buds Throughout Floral Induction

**Figure 4:**
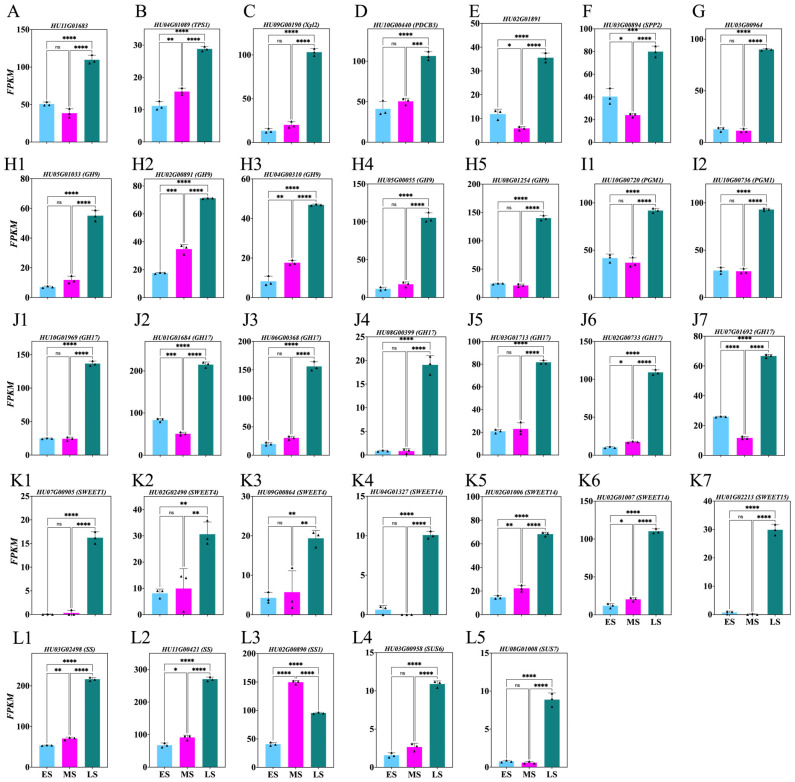
Changes in sugar synthesis genes in *Hylocereus polyrhizus* buds during the flower induction period (**A**–**L5**). The stages of flower bud induction are represented by the early stage (ES), middle stage (MS), and late stage (LS). Data are presented as mean ± SD for three replicates (*n* = 3). Small triangles represent the distribution of data for each biological repeat. Significance levels are denoted as follows: * *p* < 0.05; ** *p* < 0.01; *** *p* < 0.001; **** *p* < 0.0001; nonsignificant differences are indicated by ‘ns’ (*p* > 0.05).

To explore sugar’s role during the ES, MS, and LS of FB induction, the genes involved in starch and sugar metabolism were identified from DEGs with *p* > 0.05 (Figure 4; Supplementary Table S5). This analysis revealed a significant upregulation of the *HU11G01683*, *HU04G01089*, *HU09G00190*, *HU10G00440*, *HU02G01891*, *HU03G00894*, *HU03G00964*, *HU05G01033*, *HU02G00891*, *HU04G00310*, *HU05G00055*, *HU08G01254*, *HU10G00720*, *HU10G00736*, *HU10G01969*, *HU01G01684*, *HU06G00368*, *HU08G00399*, *HU03G01713*, *HU02G00733*, *HU07G01692*, *HU07G00905*, *HU02G02490*, *HU09G00864*, *HU04G01327*, *HU02G01006*, *HU02G01007*, *HU01G02213*, *HU03G02498*, *HU11G00421*, *HU03G00958*, *HU08G01008* genes during the LS compared to the ES and MS, while the gene *HU02G00890* was upregulated in the MS, followed by the LS and ES (Figure 4A–L).

### 2.5. Alterations in Hormone Synthesis and Signaling Pathways in Pitaya Buds Throughout Floral Induction

**Figure 5:**
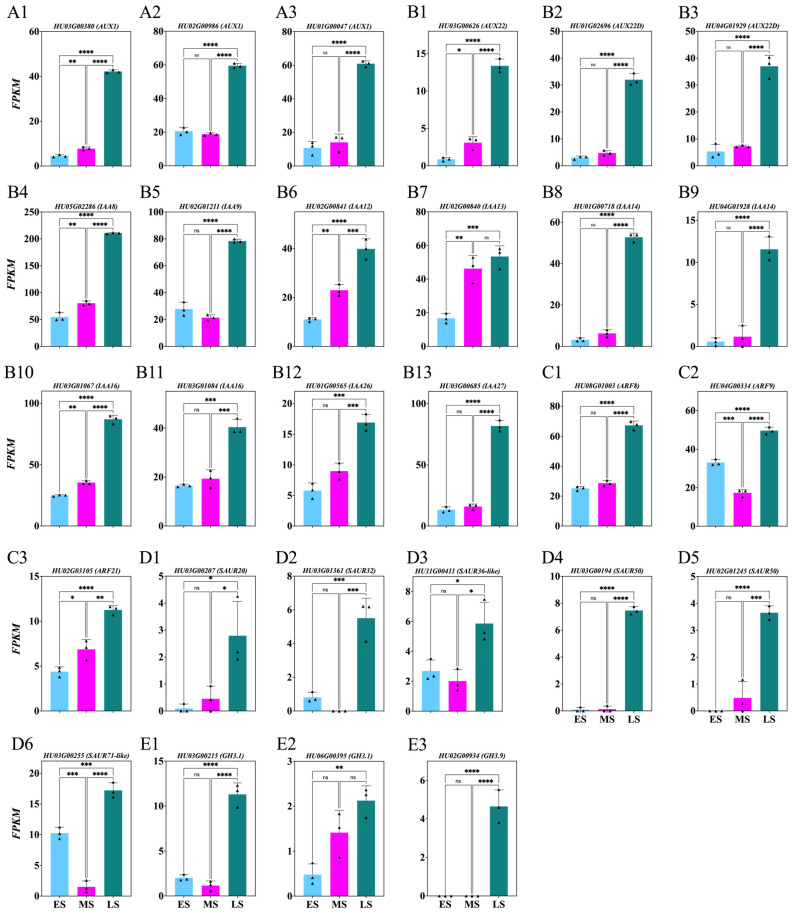
Changes in auxin synthesis and response genes in *Hylocereus polyrhizus* buds during the flower induction period (**A1**–**E3**). The stages of flower bud induction are represented by the early stage (ES), middle stage (MS), and late stage (LS). Data are presented as mean ± SD for three replicates (*n* = 3). Small triangles represent the distribution of data for each biological repeat. Significance levels are denoted as follows: * *p* < 0.05; ** *p* < 0.01; *** *p* < 0.001; **** *p* < 0.0001; nonsignificant differences are indicated by ‘ns’ (*p* > 0.05).

To explore AUX’s role during the ES, MS, and LS of FB induction, the genes involved in AUX synthesis and response were identified from DEGs with *p* > 0.05 (Figure 5; Supplementary Table S6). Significant upregulation was observed in the *HU03G00380*, *HU02G00986*, *HU01G00047*, *HU03G00626*, *HU01G02696*, *HU04G01929*, *HU05G02286*, *HU02G01211*, *HU02G00841*, *HU01G00718*, *HU04G01928*, *HU03G01067*, *HU03G01084*, *HU01G00565*, *HU03G00685*, *HU08G01003*, *HU04G00334*, *HU02G03105*, *HU03G00207*, *HU03G01361*, *HU11G00411*, *HU03G00194*, *HU02G01245*, *HU03G00255*, *HU03G00215*, *HU02G00934* genes during the LS, followed by the MS and ES (Figure 5A–E). Additionally, genes *HU02G00840* and *HU06G00395* were upregulated in the LS and MS compared to ES (Figure 5(B7,E2)).

**Figure 6:**
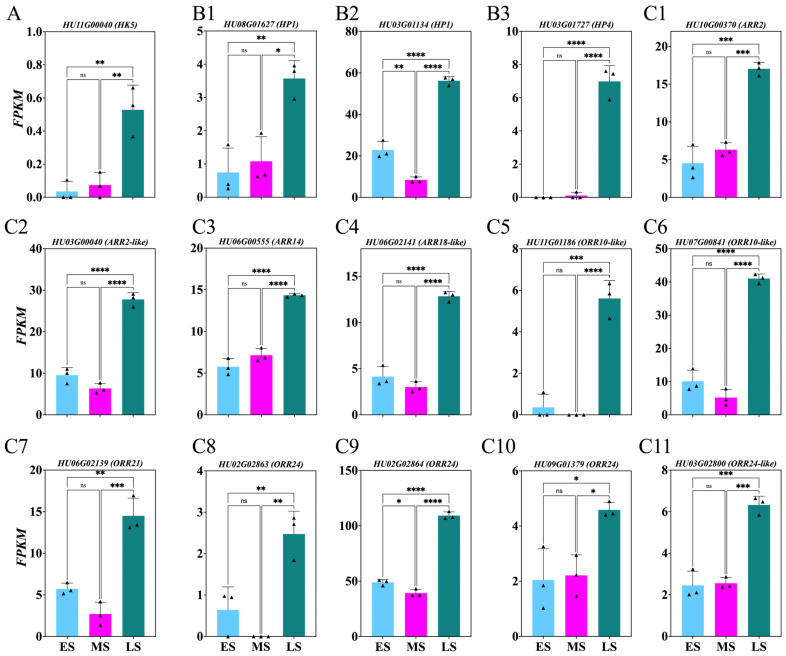
Changes in cytokinin-related genes involved in *Hylocereus polyrhizus* buds during the flower induction period (**A**–**C11**). The stages of flower bud induction are represented by the early stage (ES), middle stage (MS), and late stage (LS). Data are presented as mean ± SD for three replicates (*n* = 3). Small triangles represent the distribution of data for each biological repeat. Significance levels are denoted as follows: * *p* < 0.05; ** *p* < 0.01; *** *p* < 0.001; **** *p* < 0.0001; nonsignificant differences are indicated by ‘ns’ (*p* > 0.05).

To explore CTK’s role during the ES, MS, and LS of FB induction, the genes involved in CTK synthesis and response were identified from DEGs with *p* > 0.05 (Figure 6; Supplementary Table S7). Significant upregulation was observed in the *HU11G00040*, *HU08G01627*, *HU03G01134*, *HU03G01727*, *HU10G00370*, *HU03G00040*, *HU06G00555*, *HU06G02141*, *HU11G01186*, *HU07G00841*, *HU06G02139*, *HU02G02863*, *HU02G02864*, *HU03G02800*, *HU09G01379* genes during the LS compared with the MS and ES (Figure 6A–C).

**Figure 7:**
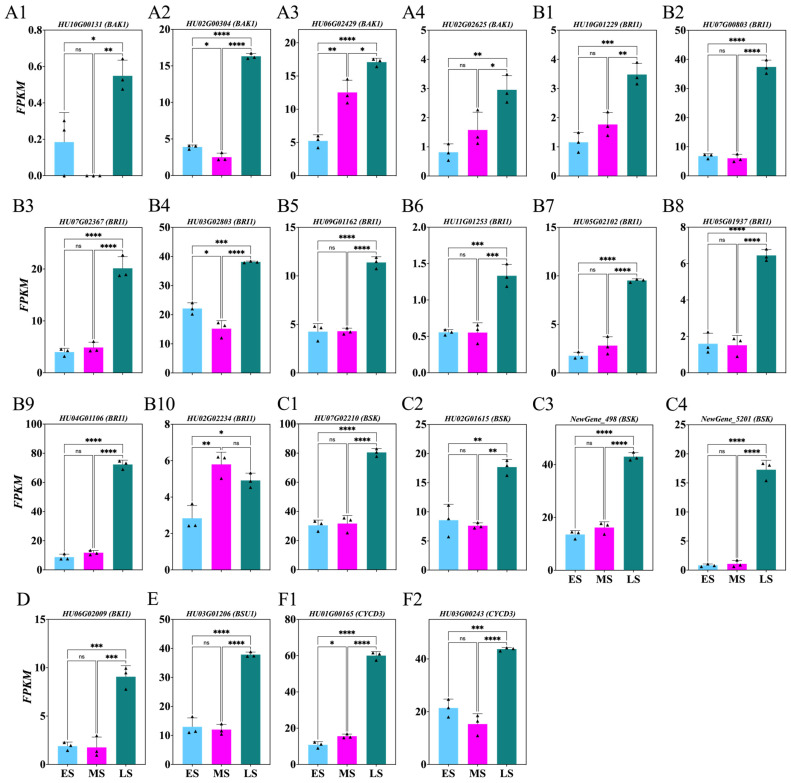
Changes in brassinosteroid-related genes involved in *Hylocereus polyrhizus* buds during the flower induction period (**A1**–**F2**). The stages of flower bud induction are represented by the early stage (ES), middle stage (MS), and late stage (LS). Data are presented as mean ± SD for three replicates (*n* = 3). Small triangles represent the distribution of data for each biological repeat. Significance levels are denoted as follows: * *p* < 0.05; ** *p* < 0.01; *** *p* < 0.001; **** *p* < 0.0001; nonsignificant differences are indicated by ‘ns’ (*p* > 0.05).

To explore BR’s role during the ES, MS, and LS of FB induction, the genes involved in BR synthesis and signaling were identified from DEGs with *p* > 0.05 (Figure 7; Supplementary Table S8). Significant upregulation was observed in the *HU10G00131*, *HU02G00304*, *HU06G02429*, *HU02G02625*, *HU10G01229*, *HU07G00803*, *HU07G02367*, *HU03G02803*, *HU09G01162*, *HU11G01253*, *HU05G02102*, *HU05G01937*, *HU04G01106*, *HU07G02210*, *HU02G01615*, *NewGene_498*, *NewGene_5201*, *HU06G02009*, *HU03G01206*, *HU01G00165*, *HU03G00243* genes during the LS compared to the MS and ES. Additionally, *HU02G02234* was upregulated in the MS and LS compared to the ES (Figure 7(B10)).

**Figure 8:**
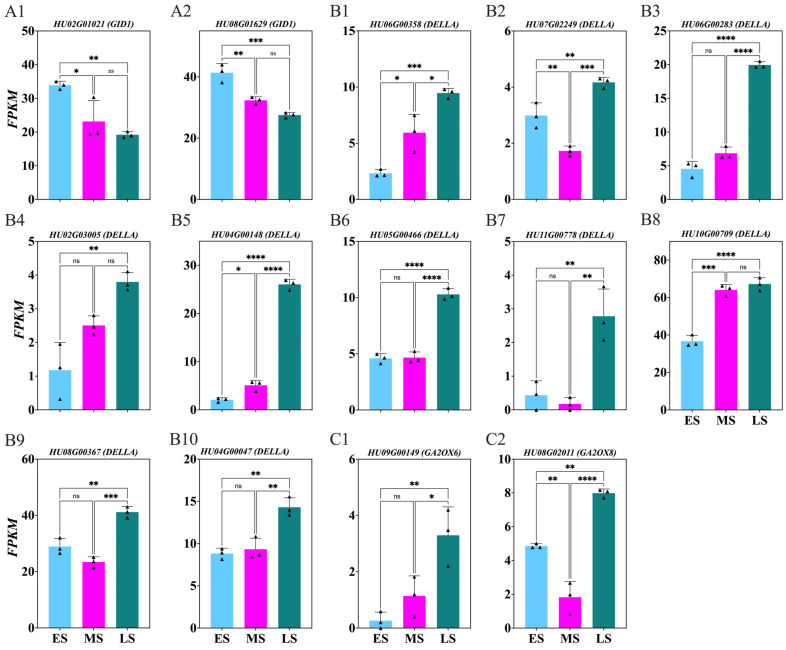
Changes in gibberellin receptor, negative regulators and oxidative degradation enzyme genes involved in *Hylocereus polyrhizus* buds during the flower induction period (**A1**–**C2**). The stages of flower bud induction are represented by the early stage (ES), middle stage (MS), and late stage (LS). Data are presented as mean ± SD for three replicates (*n* = 3). Small triangles represent the distribution of data for each biological repeat. Significance levels are denoted as follows: * *p* < 0.05; ** *p* < 0.01; *** *p* < 0.001; **** *p* < 0.0001; nonsignificant differences are indicated by ‘ns’ (*p* > 0.05).

To explore GA’s role during the ES, MS, and LS of FB induction, the genes involved in GA receptors, inhibitors, and oxidative degradation were identified from DEGs with *p* > 0.05 (Figure 8; Supplementary Table S9). Significant downregulation of the GA receptor *HU02G01021*, *HU08G01629* genes was observed in the LS compared to the MS and ES (Figure 8(A1,A2)). Conversely, the negative regulatory genes of GA signaling *HU06G00358*, *HU07G02249*, *HU06G00283*, *HU02G03005*, *HU04G00148*, *HU05G00466*, *HU11G00778*, *HU10G00709*, *HU08G00367*, *HU04G00047* were upregulated in the LS (Figure 8(B1–B10)). Additionally, the *GA2OX2* isoform genes *HU09G00149* (*GA2OX6*) and *HU08G02011* (*GA2OX8*) were upregulated in the LS compared to the MS and ES (Figure 8(C1,C2)).

### 2.6. Differential Expression of Transcription Factors in Pitaya Buds During Floral Induction

**Figure 9:**
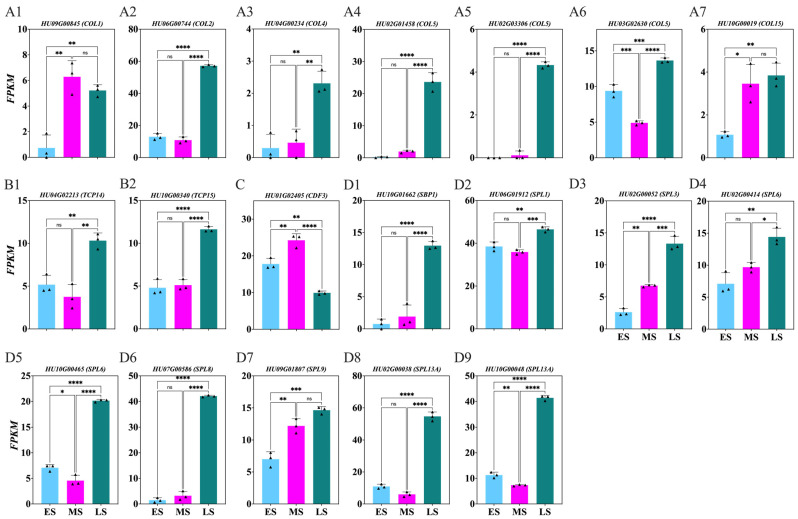
Changes in transcription factor genes involved in *Hylocereus polyrhizus* buds during the flower induction period (**A1**–**D9**). The stages of flower bud induction are represented by the early stage (ES), middle stage (MS), and late stage (LS). Data are presented as mean ± SD for three replicates (*n* = 3). Small triangles represent the distribution of data for each biological repeat. Significance levels are denoted as follows: * *p* < 0.05; ** *p* < 0.01; *** *p* < 0.001; **** *p* < 0.0001; nonsignificant differences are indicated by ‘ns’ (*p* > 0.05).

Our research identified important TF genes implicated in the photoperiod (e.g., *CONSTANS-LIKE* (*COL*), *cyclic dof factor* (*CDF*), and *TCP family transcription factor* (*TCP*)) and age or autonomous (e.g., *squamosa promoter-binding-like* (*SPL*)) flowering pathways (Figure 9; Supplementary Table S10). Significant upregulation of *COL HU06G00744*, *HU04G00234*, *HU02G01458*, *HU02G03306*, and *HU03G02630* was observed in the LS compared to the MS and ES (Figure 9(A2–A6)), while *HU09G00845* and *HU10G00019* were upregulated in the LS and MS compared to the ES (Figure 9(A1,A7)). Additionally, *TCPs* like *HU04G02213* and *HU10G00340* (Figure 9(B1–B2)) were upregulated, while *HU01G02405* (*CDF*) (Figure 9C) was downregulated in the LS compared to the MS and ES. Additionally, significant upregulation of the *SPL HU10G01662*, *HU06G01912*, *HU02G00052*, *HU02G00414*, *HU10G00465*, *HU07G00586*, *HU09G01807*, *HU02G00038*, and *HU10G00048* genes were observed in the LS compared to the MS and ES (Figure 9(D1–D9)).

### 2.7. Varied Expression of Flowering-Related Genes in Pitaya Buds Throughout the Floral Induction Phase

**Figure 10:**
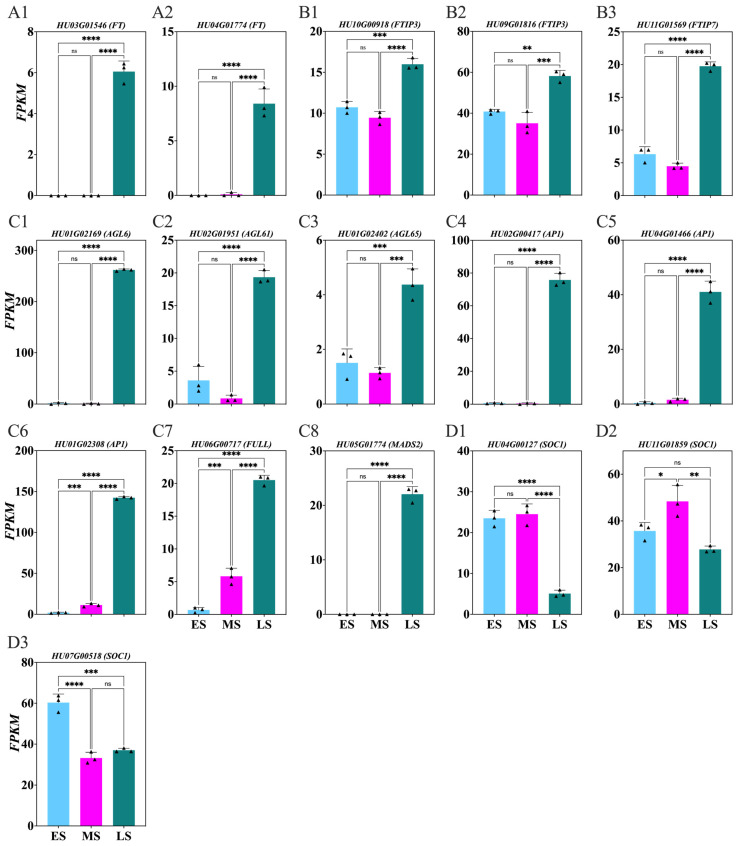
Changes in key flowering genes involved in *Hylocereus polyrhizus* buds during the flower induction period (**A1**–**D3**). The stages of flower bud induction are represented by the early stage (ES), middle stage (MS), and late stage (LS). Data are presented as mean ± SD for three replicates (*n* = 3). Small triangles represent the distribution of data for each biological repeat. Significance levels are denoted as follows: * *p* < 0.05; ** *p* < 0.01; *** *p* < 0.001; **** *p* < 0.0001; nonsignificant differences are indicated by ‘ns’ (*p* > 0.05).

The current study identified key structural genes related to flowering (Figure 10; Supplementary Table S11). Significant upregulation of the *HU03G01546*, *HU04G01774* (*FT*) (Figure 10(A1,A2)), *HU10G00918*, *HU09G01816*, and *HU11G01569 FT-INTERACTING PROTEINS* (*FTIP*) (Figure 10(B1–B3)), as well as the *HU01G02169*, *HU02G01951*, *HU01G02402*, *HU02G00417*, *HU04G01466*, *HU01G02308*, *HU06G00717*, and *HU05G01774 Agamous* (*AGL*) (Figure 10(C1–C8)) genes during the LS compared to the MS and ES was observed. Conversely, (*SOC1*) *HU04G00127*, *HU11G01859*, and *HU07G00518* were downregulated in the LS compared to the MS and ES (Figure 10(D1–D3)).

qRT-PCR analysis validated the expression of eight genes, including four TFs and four structural flowering genes, aligning with FPKM values from the pitaya transcriptome (Figure S1; Supplementary Tables S10 and S11).

## 3. Discussion

During flowering, resources, particularly sugars, are reallocated, with hormones modulating this metabolic activity. This stage involves specific gene regulation, integrating metabolic and developmental processes.

### 3.1. Dynamic Changes in Sugar Contents During Flower Induction Stages

Strigolactone, AUX, and CTK were initially believed to be regulators of bud growth [28,29]. However, later studies found that sugar demand, not AUX, is the master regulator of apical buds and the release of an active form of hormone that may act after [30], highlighting the importance of sugar in FB formation. Sugar signal is rapidly communicated and triggers growth. *HU11G01683* (Figure 4A) is an ectonucleotide pyrophosphatase/phosphodiesterase enzyme gene, involved in the nucleotide hydrolysis, leading to sugar formation [31]. *HU04G01089* (Figure 4B), encoding trehalose-phosphate synthase 1 (*TPS1*), catalyzes trehalose synthesis, a disaccharide sugar [32,33] and influences flowering time in Arabidopsis [15]. *HU09G00190* (Figure 4C), a β-xylosidase/α-L-arabinofuranosidase 2 (*Xyl2*), breaks down complex sugars in plant cell walls [34] and is regulated during flowering in Arabidopsis [35]. *HU10G00440* (Figure 4D), a plasmodesmata callose-binding protein 3 (*PDCB3*), regulates the transport of sugars, hormones, and *FT* signaling, playing a key role in phase change [36,37]. *HU02G01891* (Figure 4E), a *phosphofructokinase* (*PFK7*) gene, is involved in fructose metabolism and early flowering in Arabidopsis [38]. *HU03G00894* (Figure 4F), encoding *sucrose-phosphatase 2* (*SPP2*), is vital for plant energy during the FB formation period [39]. *HU03G00964* (Figure 4G) is involved in the synthesis of UDP-glucose, necessary for cell wall polysaccharides influencing flowering [40]. The genes *HU05G01033*, *HU02G00891*, *HU04G00310*, *HU05G00055*, and *HU08G01254* (Figure 4(H1–H5)) from glycosyl hydrolase family 9 (*GH9*) play a role in cellulose synthesis by breaking down starch into glucose. In wheat, *TaGH9* significantly affects pollen sterility and anther opening [41], while in Arabidopsis *AtCEL1,* a homologue of *HU08G01254* has shown increased yield in *Setaria viridis*, indicating *GH9*’s role in flowering [42]. Sugar molecules attach to several plant hormones to form glycosides. AUX, GA3, and CTK may be released when glycosidic linkages are hydrolyzed by glycosyl hydrolases, potentially contributing to flowering [43]. *HU10G01969*, *HU01G01684*, *HU06G00368*, *HU08G00399*, *HU03G01713*, *HU02G00733*, *HU07G01692* are *glycosyl hydrolases family 17* (*GH17*) genes (Figure 4(J1–J7)), involved in hydrolyzing β-1,3-glucans, modifying and degrading callose, indirectly influencing flowering. In *Populus*, chilling triggers the upregulation of *FT* and GA-responsive *GH17* enzymes, which are crucial for cell wall remodeling and the transport of signals necessary for flowering [37]. *HU10G00720*, *HU10G00736* (Figure 4(I1,I2)), encoding phosphoglucomutase (*PGM1*), convert glucose-1-phosphate to glucose-6-phosphate for starch synthesis leading to ADP-glucose and energy production [44]. Additionally, the genes *HU07G00905*, *HU02G02490*, *HU09G00864*, *HU04G01327*, *HU02G01006*, *HU02G01007*, *HU01G02213* (Figure 4(K1–K7)) encode (*SWEET*) sugar transporters, while *HU03G02498*, *HU11G00421*, *HU02G00890*, *HU03G00958*, and *HU08G01008* (Figure 4(L1–L5)) are involved in sucrose synthesis (*SS*). In Arabidopsis, overexpression of *SWEET10* can upregulate FT expression and accelerate flowering [45]. The *Jatropha curcas JcSWEET16* gene, when overexpressed in Arabidopsis, modifies the flowering time [46]. Overexpression of *SS 006G213100* sorghum gene in Arabidopsis shortens the flowering time [47]. Overall, these findings indicate that starch and sugar metabolism genes are crucial for phase transition and provide necessary energy during FB’s formation in pitaya.

### 3.2. Dynamic Changes in Hormone Levels During Flower Induction Stages

Hormones significantly influence the developmental trajectories of FB formation. AUX gradients are crucial for floral organ morphogenesis, including sepals, petals, stamens, and carpels. AUX interacts with other phytohormones, providing nuanced control over floral development. AUX can downregulate GA and facilitate the flowering process in long-day plants [48]. The AUX-CTK equilibrium modulates the transition from vegetative to floral meristems [49]. Auxin influences gene expression crucial for flower development, affecting both its own signaling components and other hormonal pathways. *HU03G00380*, *HU02G00986*, and *HU01G00047* are auxin influx carriers (*AUX1*) (Figure 5(A1–A3)) that facilitate AUX entry into cells and mediate flowering [50]. Mutations in these carriers alter AUX transport, impacting flowering genes involved in flower organ identity and meristem integrity [51]. *HU03G00626*, *HU01G02696*, *HU04G01929*, *HU05G02286*, *HU02G01211*, *HU02G00841*, *HU02G00840*, *HU01G00718*, *HU04G01928*, *HU03G01067*, *HU03G01084*, *HU01G00565*, and *HU03G00685* are auxin-responsive proteins (*AUX/IAA*) (Figure 5(B1–B13)), key players in the AUX signaling pathway. Overexpression of *TaIAA15–1A* caused early flowering by interacting with *BdARF16* in *Brachypodium distachyon* [52]. *HU08G01003*, *HU04G00334*, and *HU02G03105* are *ARF* (Figure 5(C1–C3)), where mutations in *arf2* delay flowering in Arabidopsis [53]. *HU03G00255*, *HU03G00207*, *HU03G01361*, *HU03G00194*, *HU02G01245*, and *HU11G00411* are *Small auxin-upregulated RNAs* (*SAURs*) (Figure 5(D1–D6)), mainly responding to light and high temperature, and affecting floral organ size [54,55,56]. *HU03G00215*, *HU06G00395*, and *HU02G00934* represent auxin-responsive GH3 family genes (Figure 5(E1–E3)), crucial in early AUX homeostasis and potentially influencing flowering through AUX signal modulation [57]. CTK influences SAM activity and floral meristem formation, influencing bud fate toward vegetative or floral structures based on CTK and AUX interplay [21]. CTK modulates flowering timing through interactions with floral regulatory genes [58] and is essential for floral organ differentiation, including petals and stamens development [59]. *HU11G00040* is *histidine kinase* (*HK*) (Figure 6A), key to CTK signal transduction, particularly influencing plant organ development and flowering time. Mutations in *AHK2* and *AHK3* underscore their roles in modulating organ size and flowering time. *HU08G01627*, *HU03G01134*, *HU03G01727* are histidine phosphotransfer proteins (*HP*) (Figure 6(B1–B3)), while *HU10G00370*, *HU03G00040*, *HU06G00555*, *HU06G02141*, *HU11G01186*, *HU07G00841*, *HU06G02139*, *HU02G02863*, *HU02G02864*, *HU03G02800*, and *HU09G01379* are CTK response regulators (*ARR-A* and *ARR-B* family) (Figure 6(C1–C11)), involved in CTK signaling and possibly influencing flowering. Brassinosteroids modulate cellular processes like elongation, division, and differentiation, pivotal for flowering. They are sensed by the *BRI1* receptor kinase, which downregulates *FLC* expression, facilitating floral transition [60]. BRs also coordinate with other hormones to regulate key floral identity genes, including *LFY* and *AP1* [61,62]. *HU10G00131*, *HU02G00304*, *HU06G02429*, and *HU02G02625* are *BRASSINOSTEROID INSENSITIVE 1-associated receptor kinase 1* (*BAK1*) (Figure 7(A1–A4)), which, together with *BRI1*, regulate sugar and promote flowering [63]. *HU10G01229*, *HU07G00803*, *HU07G02367*, *HU03G02803*, *HU09G01162*, *HU11G01253*, *HU05G02102*, *HU05G01937*, *HU04G01106*, and *HU02G02234* are *BRASSINOSTEROID INSENSITIVE 1* (*BRI1*) genes (Figure 7(B1–B10)). Mutations in *BRI1* delay flowering due to altered endogenous BR levels [64], and overexpression induces early flowering [65]. *HU07G02210*, *HU02G01615*, *NewGene_498*, and *NewGene_5201* are *BR-signaling kinase* (*BSK*) (Figure 7(C1–C4)), functioning upstream of *BIN2* and downstream of *BRI1*, contributing to BR signaling and influencing flowering. *HU06G02009* is *BRI1 kinase inhibitor 1* (*BKI1*) (Figure 7D), which negatively regulates *BRI1.* Mutations in *BKI1* can impact the BR pathway [66]. *HU03G01206* is a serine/threonine protein phosphatase (*BSU1*) (Figure 7E) involved in *BES1* phosphorylation and *BIN2* inhibition, playing roles in cell growth and expansion, potentially related to flowering [67]. *HU01G00165*, and *HU03G00243* are *Cyclin-D3-1 (CYCD3)* genes (Figure 7(F1,F2)), regulating the G1-to-S phase transition in the cell cycle and crucial for CTK responses and BR signaling. Mutations in *CYCD3;1* affect SAM activity and reduce CTK responses, impacting flowering timing [68]. Gibberellins can inhibit flowering genes under non-inductive photoperiods [69] and prioritize survival over reproduction during environmental stress [70]. DELLA genes, GA signaling repressors, alter binding to the SOC1 gene, affecting flowering timing. GA-mediated *DELLA* degradation increases flowering [71]. Pitaya GA signaling was downregulated during flower induction, indicated by the downregulation of the GA receptor genes *HU02G01021*, *HU08G01629*, and *GID1* (Figure 8(A1,A2)), and upregulation of the GA negative regulators *HU06G00358*, *HU07G02249*, *HU06G00283*, *HU02G03005*, *HU04G00148*, *HU05G00466*, *HU11G00778*, *HU10G00709*, *HU08G00367*, and *HU04G00047* (*DELLA*) (Figure 8(B1–B10)). Additionally, the upregulation of GA oxidative degradation enzyme *HU09G00149* (*GA2OX6*) and *HU08G02011* (*GA2OX8*) (Figure 8(C1,C2)) supported decreased GA levels, downregulated *GID1*, and upregulated *DELLA* and *GA2OX* genes.

### 3.3. Key TF Genes During Flower Induction Stages

Critical TF genes involved in the photoperiod (e.g., *COL*, *CDF*, *TCP*) and age/autonomous (e.g., *SPL*) flowering pathways were identified. *HU09G00845*, *HU06G00744*, *HU04G00234*, *HU02G01458*, *HU02G03306*, *HU03G02630*, and *HU10G00019* encode *COL* genes (Figure 9(A1–A7)), which act through the photoperiod pathway. In Arabidopsis, overexpression of *COL5* regulates early flowering [72]. In tomato, *SlCOL*, *SlCOL4a*, and *SlCOL4b* trigger key flowering genes [73]. In cotton, *GhCOL1-A* and *GhCOL1-D* induce flowering [74]. In soybean, mutations in *GmCOL1a* and *GmCOL1b* affect flowering time [75]. *HU04G02213*, *HU10G00340* are *TCP* (Figure 9(B1,B2)), involved in regulating flowering through the photoperiod pathway. In Arabidopsis, *TCP4* binds to *GIGANTEA* (*GI*) and induces *CO* expression [76]; also, *TCP7* activate SOC1 expression upon interacting with *CO* [77]. *HU01G02405* is a *CDF* gene (Figure 9C) that controls flowering by repressing the *CO* and *FT* in Arabidopsis [78].

*HU10G01662*, *HU06G01912*, *HU02G00052*, *HU02G00414*, *HU10G00465*, *HU07G00586*, *HU09G01807*, *HU02G00038*, and *HU10G00048* are *SPL* genes (Figure 9(D1–D9)) regulated by miR156. As plants age, miR156 levels decrease, increasing *SPL* levels, which promote *FT* and other genes critical for floral transition, linking developmental stage and flowering time [79]. In alfalfa, silencing *SPL13* results in late flowering [80]. In switchgrass, overexpression of *SPL7* and *SPL8* enhances flowering and sugar accumulation by directly upregulating *SEPALLATA3* (*SEP3*) and *MADS32* [81]. *SOC1* and *FT* regulate *SPL3*, *SPL4* and *SPL5* promoters in Arabidopsis in response to photoperiod signals [82]. Additionally, in Arabidopsis, *SPL10* targets the promoters of *FUL* and *LFY* gene via *MED25* [83].

### 3.4. Key Flowering Genes During Flower Induction Period

HU03G01546 and HU04G01774 (Figure 10(A1,A2)) are FT genes regulated by the photoperiod-responsive gene CO [84]. FT is transported from leaves to the SAM, where it interacts with SOC1 and FD to trigger flowering [85]. HU10G00918, HU09G01816, and HU11G01569 (Figure 10(B1–B3)) are FTIP1 genes, critical for transporting FT to the meristem for floral transition [86]. Mutations in FTIP1 disrupt this transport, impacting flowering timing, while the loss of FTIP3 is associated with reduced flowering [87]. HU01G02169, HU02G01951, HU01G02402, HU02G00417, HU04G01466, HU01G02308, HU06G00717, and HU05G01774 are AGL genes (Figure 10(C1–C8)), essential for flower development and specifying the identities of petals, stamens, carpels, and meristems [88]. LEAFY (LFY) promotes AGL gene activity, determining stamen and carpel identity [89]. HU04G00127, HU11G01859, and HU07G00518 (Figure 10(D1–D3)) are SUPPRESSOR OF OVEREXPRESSION OF CONSTANS 1 (SOC1) genes, activated by CO in the photoperiod pathway, SPL9, through age-dependent pathways [90], and FLC by the vernalization pathway [91]. FT primarily activates AP1, essential for flower meristem identity, by activating the A function of ABC model and inhibiting flowering time genes like SOC1, AGL24, and SVP [90]. SOC1 is required to induce flowering, but, after flowering, it is confirmed it is downregulated in buds by AP1 to secure floral reversion.

**Figure 11:**
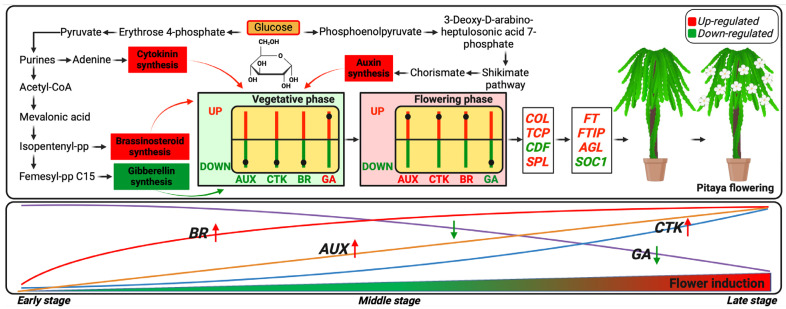
Schematic representation of the regulatory mechanisms through which sugar and hormone signaling activate key transcription factors (TFs) and floral genes in pitaya flowering.

In this study, molecular determinants of pitaya during the flower induction period were identified. Firstly, glucose serves as a basic precursor in the shikimate pathway, leading to the production of tryptophan and chorismate, which are critical for AUX synthesis during the flowering induction period. Secondly, glucose metabolism, specifically through glycolysis and the pentose phosphate pathway, facilitates the synthesis of active CTK during this period. Thirdly, glycolysis of glucose produces acetyl-CoA, which enters the mevalonate pathway to produce mevalonic acid, ultimately leading to the synthesis of BR during the FB induction period. Fourth, a metabolic shift in these pathways during the flower induction period results in decreased levels of GA, which is crucial for FB induction in pitaya (Figure 11).

## 4. Materials and Methods

### 4.1. Experimental Site and Climatic Condition

The current experiment was conducted in germplasm and a resource garden for pitaya, South China Agricultural University, Guangzhou, China (23.158′ N, 113.361′ E). Guangzhou has subtropical weather with mild winters and hot, rainy, humid summers. Monthly meteorological data were obtained from an online website (https://www.weather-atlas.com/en/china/guangzhou-climate; accessed on 20 January 2024). Daylight hours were converted to decimals using Furey, Edward’s “Time to Decimal Calculator” (https://www.calculatorsoup.com/calculators/time/time-to-decimal-calculator.php; accessed on 20 January 2024) from CalculatorSoup. Monthly climatic data are available in Supplementary Table S1.

### 4.2. Experimental Design and Sampling

Plant material was collected from 60 six-year-old pitaya plants. Two buds per plant were sampled on 8, 18, and 28 April 2023, resembling the early stage (ES), middle stage (MS), and late stage (LS), respectively. The samples were brought on ice to the laboratory. Extra green and dead brown tissues were removed with a clean razor. Bud samples with three replicates at the ES, MS, and LS were separately ground into powder using liquid nitrogen with a rapid grinder (Tissue-lyser-24, Shanghai Jingxin Industrial Development Co., Ltd., Shanghai, China) and then subjected to sugar and hormone analyses. The remaining sample was stored at −80 °C for RNA sequencing.

### 4.3. Extraction and Determination of Soluble Sugar

A 0.3 g sample was homogenized with 6 mL of 90% ethanol in a 10 mL tube, heated in a water bath at 80 °C for 20 min, and centrifuged at 4000× *g* rpm for 10 min. The supernatant was decanted into a 15 mL glass tube. The residue was re-extracted with 4 mL of 90% ethanol using the same protocol. The combined extracts were dried using a Rapid VAP system. The dried sample was reconstituted with 2 mL of ultra-pure water, swirled for 1 min, transferred to a 2 mL tube, and centrifuged at 13,000× *g* rpm for 10 min. The supernatant was processed through a Sep-Pak 1cc (100 mg) C18 cartridge (Beijing Yunpeptide Biotechnology Co., Ltd. Beijing, China) and filtered through 0.45 μm tips into a black HPLC vial. Calibration standards were prepared by dissolving 0.1 g each of sucrose, fructose, and glucose in 100 mL of ultra-pure water, with 1 mL of this solution transferred to a black HPLC vial using 0.45 μm tips [92]. Post-derivatization, both samples and standards were analyzed using an Agilent 1200LC High-Performance Liquid Chromatography system (Agilent Technology, Palo Alto, CA, USA) to quantify the metabolites.

### 4.4. Measurements of Total Sugar and Starch

Bud samples (0.5 g) were extracted with 4 mL of 80% methanol in a 15 mL centrifuge tube and incubated in a water bath at 80 °C for 30 min. After centrifugation at 5650× *g* rpm for 10 min, the supernatants were transferred to a 50 mL centrifuge tube. The residues were re-extracted twice with 2 mL of 80% ethanol each, following the same procedure. Activated carbon (10 mg) was added to the combined supernatants and incubated at 80 °C for 30 min. The supernatants were diluted to 25 mL with distilled water and used to measure total sugar. The residues were dried at 80 °C, gelatinized by adding 2 mL of distilled water and boiling for 10 min, and then cooled in ice water. Subsequently, 2 mL of 9.2 M perchloric acid and 6 mL of distilled water were added and mixed thoroughly. After centrifugation, the supernatant was transferred to a 25 mL volumetric flask. The previous steps were repeated with 4.6 M perchloric acid. For starch measurement, 1 mL of supernatant was mixed with 5 mL of anthrone (1 μg/mL), boiled for 10 min, and absorbance was measured at 620 nm using a spectrophotometer.

### 4.5. Plant Hormone Quantification

Three replicates of the ES, MS, and LS buds ground samples (100 mg each) were placed in 2 mL centrifuge tubes. Each sample was mixed with 1 mL of ethyl acetate, shaken at 2000 rpm for 10 min, and then centrifuged at 12,000× *g* rpm for 10 min. The supernatant was moved to a new 2 mL centrifuge tube, and the organic solvent was evaporated by a nitrogen blower. The dried samples were reconstituted with 200 μL of 50% methanol, centrifuged at 13,000× *g* rpm for 15 min, and the supernatant filtered through a 0.22 μm organic filter membrane. Finally, 100 μL of the filtered supernatant was transferred to a sample vial for chromatographic analysis. For quantification, authentic reference standards of AUX, dhZR, ZR, iPA, BR, GA3, and GA4 (all from Yuanye Biotech, Shanghai, China) were used. Hormones were analyzed by an Agilent 1290 Infinity II-6470 system with a ZORBAX Eclipse Plus C18 column (1.8 μm, 2.1 × 50 mm^2^) (Agilent Technologies, Santa Clara, CA, USA). The mobile phase was composed of (A) 0.1% (*v*/*v*) formic acid and (B) methanol, applying a gradient program that included an isocratic phase of 75% A and 25% B for 0–0.8 min, followed by a linear gradient from 75% to 25% A and 25% to 75% B from 0.8 to 1.5 min, and concluding with a linear gradient from 25% to 75% A and 75% to 25% B from 1.5–2.5 min, with a flow rate of 0.3 mL/min. A sample volume of 5 μL was injected for analysis. GA3 and GA4 were detected using an electrospray ionization source in the negative ionization mode, while AUX was detected in the positive mode. Dynamic multiple reaction monitoring was optimized for each metabolite based on the standards. Instrumental settings included a capillary voltage of 3500 V, nebulizer gas pressure of 45 psi, sheath gas flow rate of 11 L/min, and column oven temperature of 300 °C. Hormone measurements were based on extracted ion chromatograms and comparison with standard solutions.

### 4.6. RNA Quantification and Qualification

Total RNA was extracted from pitaya buds (three biological replicates each for the ES, MS, and LS) using the RNAprep Pure Plant Kit (Tiangen, Beijing, China) following the manufacturer’s instructions. RNA purity, concentration, and integrity were assessed using NanoDrop, Qubit 2.0 (Thermo Fisher Scientific Inc., Waltham, MA, USA), and Agilent 2100 Bioanalyzer (Agilent Technologies, Santa Clara, CA, USA).

### 4.7. Library Construction and RNA Deep Sequencing

For RNA sample preparations, 1 μg of RNA per sample was utilized. Sequencing libraries were created using the NEBNext Ultra RNA Library Prep Kit for Illumina (New England Biolabs GmbH, Frankfurt, Germany) according to the manufacturer’s guidelines. Index codes were assigned to each sample for sequence identification. mRNA was isolated from total RNA using poly-T oligo-attached magnetic beads and then fragmented with divalent cations at high temperature. First-strand cDNA was synthesized using random hexamer primer and M-MuLV Reverse Transcriptase, followed by second-strand cDNA synthesis with DNA Polymerase I and RNase H. The resulting overhangs were blunted, adenylated at the 3′ ends, and ligated with NEBNext Adaptor. The cDNA fragments (~240 bp) were purified using the AMPure XP system (Beckman Coulter, Beverly, CA, USA). After treatment with 3 μL USER Enzyme (New England Biolabs GmbH, Frankfurt, Germany) at 37 °C for 15 min and 95 °C for 5 min, PCR amplification was carried out using Phusion High-Fidelity DNA polymerase, Universal PCR primers, and Index (X) Primer. The final PCR products were purified using the AMPure XP system, and library quality was evaluated with an Agilent Bioanalyzer 2100 system.

### 4.8. Transcriptome Analysis Using Reference Genome-Based Reads Mapping

Raw data were processed using custom Perl scripts to remove adapters, poly-N sequences, and low-quality reads, resulting in clean reads. Quality metrics, including Q20, Q30, GC content, and duplication rates, were calculated. The clean reads were then aligned to the pitaya genome (http://pitayagenomic.com/; accessed on 1 January 2024) using Hisat2 [93]. Gene expression levels were quantified as FPKM values (fragments per kilobase of exon per million fragments mapped) with Cufflinks software (Version 1.3.0) [94].

### 4.9. Identification of Differential Gene Expression

DEG analysis for the ES, MS and LS was conducted using DESeq2. *p*-values were adjusted by Benjamini and Hochberg’s method to control the false discovery rate. Genes with an adjusted *p*-value of less than 0.01 were deemed DEGs. KOBAS software (Version 2.0) [95] was employed to assess the statistical enrichment of these DEGs in the Kyoto Encyclopedia of Genes and Genomes (KEGG) pathways.

### 4.10. Sequence Annotation

The genes were compared to various protein database using BLASTX, including the National Center for Biotechnology Information (NCBI) database, NCBI non-redundant protein sequences (Nr), Pfam (Protein family), Swiss-Prot (a manually annotated and reviewed protein sequence database), and the KO (KEGG Ortholog) database, with a cut-off E-value of 10^−5^. The genes were identified based on the best BLAST hit (highest score) and their corresponding protein functional annotation.

### 4.11. qRT-PCR Validation

For quantitative real-time PCR (qRT-qPCR) validation, eight genes were selected from the DEG list (four related to TF signaling and four to flowering). Primers were designed using NCBI Primer-BLAST (https://www.ncbi.nlm.nih.gov/tools/primer-blast/; accessed on 10 January 2024) (Supplementary Table S2). qRT-PCR analyses were conducted on a CFX384 Real-Time System (Bio-Rad, Irvine, CA, USA), using RealUniversal Color PreMix (SYBR Green) TIANGEN (Beijing, China). The pitaya ACTIN gene (*HU07G00802*) served as the internal standard. Expression levels were analyzed in three replications using the 2^−ΔΔC^_T_ method [96].

### 4.12. Statistical Analysis

An ordinary one-way ANOVA with Šídák’s multiple comparisons test was used to compare group means. Graphpad PRISM version 9.1.1 for Mac (Graphpad Software, San Diego, CA, USA, www.graphpad.com) was used to perform the analysis. The results were presented as mean standard deviations (*n* = number of repeats), with significance values as follows: * *p* ˂ 0.05; ** *p* ˂ 0.01; *** *p* ˂ 0.001; **** *p* ˂ 0.0001; and non-significant (ns) (*p* > 0.05).

## 5. Conclusions

In summary, this study highlights the intricate regulatory network governing flower bud (FB) induction in pitaya. Our findings demonstrate a significant upregulation of sugars, starch, and phytohormones such as AUX, CTK, and BR during the LS of flower induction, accompanied by the downregulation of active gibberellins (GA3, GA4). Through a transcriptomic analysis, we identified key genes involved in starch and sugar metabolism, AUX, CTK, and BR biosynthesis, as well as GA signaling, including receptors, negative regulators, and oxidative degradation pathways. Importantly, this study reveals a complex multidimensional regulatory network encompassing 10 photoperiod-related genes (e.g., *COL*, *CDF*, and *TCP* family), 9 age or autonomous pathways (e.g., SPL), and 16 key flowering genes, including *FT*, *FTIP*, *AGL*, and *SOC1*). These findings offer deeper insights into the molecular mechanisms of pitaya flowering induction. To further contextualize these results, we developed a hypothetical model illustrating the hormonal fluctuations and sugar level adjustments critical for successful FB induction. This model provides a foundation for future research, including functional analyses of identified genes and targeted hormone applications during the induction phase. Such approaches could enhance our understanding of pitaya’s flowering mechanisms and contribute to improved cultivation practices for this economically significant crop.

## Figures and Tables

**Table 1:** KEGG pathways significantly up- and downregulate DEGs between the ES-LS pitaya buds, related to hormone signaling, starch and sugar metabolism and intracellular activity.

Pathway	Pathway ID	Gene No.	Up	Down
Plant–pathogen interaction	ko04626	201	89	112
Plant hormone signal transduction	ko04075	176	84	92
MAPK signaling pathway—plant	ko04016	144	57	87
Carbon metabolism	ko01200	119	71	48
Starch and sucrose metabolism	ko00500	116	61	55
Protein processing in endoplasmic reticulum	ko04141	93	60	33
Biosynthesis of amino acids	ko01230	90	74	16
Ribosome	ko03010	90	67	23
Phenylpropanoid biosynthesis	ko00940	85	61	24
Glycolysis/Gluconeogenesis	ko00010	81	51	30
Amino sugar and nucleotide sugar metabolism	ko00520	72	45	27
Endocytosis	ko04144	72	41	31
Pentose and glucuronate interconversions	ko00040	65	45	20
Photosynthesis	ko00195	57	3	54
Spliceosome	ko03040	54	32	22
Circadian rhythm—plant	ko04712	51	20	31
RNA transport	ko03013	49	34	15
Oxidative phosphorylation	ko00190	48	27	21
Ubiquitin-mediated proteolysis	ko04120	47	25	22
Galactose metabolism	ko00052	46	25	21
Total	-	1756 (100%)	972 (55.35%)	784 (44.64%)

## Data Availability

The datasets used and analyzed in the current study are available from the corresponding author on reasonable request. Sequences have been deposited in NCBI Sequence Read Archive under project PRJNA1143690.

## Supplementary Materials

The following supporting information can be downloaded at: https://www.mdpi.com/article/10.3390/ijms26031250/s1.
