# Supplementary material for: Transcriptome Analysis Reveals Sugar and Hormone Signaling Pathways Mediating Flower Induction in Pitaya (Hylocereus polyrhizus)
Source: Int J Mol Sci. 2025 Jan 31;26(3):1250. doi: 10.3390/ijms26031250 (PMC11818635; doi:10.3390/ijms26031250)
Supplement: Supplementary file 1 [file ijms-26-01250-s001.zip › ijms-3416373-supplementary.pdf]

Supplementary information

Transcriptome Analysis Reveal Sugar and Hormone Signaling Pathways Mediating Flower Induction in Pitaya (*Hylocereus polyrhizus*)

Kamran Shah, Xiaoyue Zhu, Zhang Tiantian, Jiayi Chen, Chen Jiaxuan, Yonghua Qin *

Table S1. Monthly meteorological statistics of experimental site.

Guangzhou, Guangdong, China	Min (°C)	Max (°C)	Precipitation days (mm)	Daylight (h)	Decimal (h)	Sunshine (h)	Humidity (%)	UV-index
January	10.3	18.3	7.5/40.9	10h 54min	10.90	3.8	72	7
February	11.7	18.5	11.2/69.4	11h 23min	11.38	2.6	78	9
March	15.2	21.6	15/84.7	12h 1min	12.01	2	82	11
April	19.5	25.7	16.3/201.2	12h 41min	12.68	2.2	84	12
May	22.7	29.3	18.3/283.7	13h 15min	13.25	3.4	84	12
June	24.8	31.5	18.2/276.2	13h 30min	13.50	4.7	84	12
July	25.5	32.8	15.9/232.5	13h 24min	13.40	6.5	82	12
August	25.4	32.7	16.8/227	12h 55min	12.91	5.6	82	12
September	24	31.5	12.5/166.2	12h 17min	12.28	5.7	78	11
October	20.8	28.8	7.1/87.3	11h 37min	11.61	5.9	72	9
November	15.9	24.5	5.5/35.4	11h 2min	11.03	5.8	66	7
December	11.5	20.6	4.9/31.6	10h 42min	10.70	5.4	66	6

Table S2. Primers used in this study.

Gene ID	Gene Name	Primer sequence (5'-3')
HU02G01458	***COL5***	F : TACACCACCAACAACATCGA<br>R : TGCTGCGATACCTCTCAATT
HU04G00234	***COL4***	F : CCCTCCTCCAAGAAATCTCC<br>R : GGCAATTTGGTTAAGCGACT
HU10G00340	***TCP15***	F : CCTCTTCCGTTGGATCATCA<br>R : TCATGATGATGGTCCTCTGC
HU07G00586	***SPL8***	F : ATGTTCCACGACCCATACTC<br>R : GGTGGTTTGGAGGTTGTAGA
HU03G01546	***FT***	F : GGCCACCAAGAGATCCATTA<br>R : TCCGATCTCCACTCTAGGTT
HU04G01774	***FT***	F : CGACTTTGTGGCTTCAACTT<br>R : CCCGGAATATCTGTCACCAA
HU01G02169	***AGL6***	F : GTGATGTTGAGATTGCCACC<br>R : GATAGTCCTGGAGTTGTGGG
HU02G00417	***AP1***	F : CTAATGCCTGCTTTCAACCC<br>R : CATGTCGACGTAAAGCATCC
HU07G00802	***Actin***	F : GTCACACGGTTCCCATCTAT<br>R : GACATAGGCAAGCTTCTCCT

Table S3. Summary of sequencing data for the clean reads in each sample and every replicate. Red and green values represent highest and lowest value, respectively in each column.

Samples	Replicate names	Clean reads	Clean bases	GC content (%)	Q20 (%)	Q30 (%)
ES	ES-1	31,882,327	9,529,859,098	49.82	96.9	92.12
	ES-2	35,604,642	10,646,806,736	49.69	97.11	92.50
	ES-3	32,812,111	9,822,148,814	49.90	96.87	92.07
MS	MS-1	36,965,573	11,061,372,806	50.61	97.2	92.79
	MS-2	35,933,381	10,751,192,332	50.66	97.23	92.83
	MS-3	33,609,102	10,048,473,262	50.68	97.44	93.30
LS	LS-1	34,886,431	10,445,172,012	47.13	97.09	92.40
	LS-2	32,428,609	9,699,530,790	47.14	97.56	93.36
	LS-3	33,298,360	9,965,609,074	47.19	97.29	92.71

Table S4. Summary of the sequencing data in each sample. Red and green values represent highest and lowest value in each column.

Samples	Replicate names	Total reads	Mapped reads	Unique mapped reads	Multiple map reads	Reads map to '+'	Reads map to '-'
ES	ES-1	63,764,654	12,055,986 (18.91%)	11,395,912 (17.87%)	660,074 (1.04%)	6,449,340 (10.11%)	6,466,970 (10.14%)
	ES-2	71,209,284	13,953,176 (19.59%)	13,188,059 (18.52%)	765,117 (1.07%)	7,467,616 (10.49%)	7,490,110 (10.52%)
	ES-3	65,624,222	12,328,954 (18.79%)	11,660,293 (17.77%)	668,661 (1.02%)	6,591,945 (10.04%)	6,613,436 (10.08%)
MS	MS-1	73,931,146	11,109,793 (15.03%)	10,529,765 (14.24%)	580,028 (0.78%)	5,927,866 (8.02%)	5,952,510 (8.05%)
	MS-2	71,866,762	10,479,819 (14.58%)	9,930,457 (13.82%)	549,362 (0.76%)	5,588,439 (7.78%)	5,613,692 (7.81%)
	MS-3	67,218,204	9,485,428 (14.11%)	8,988,185 (13.37%)	497,243 (0.74%)	5,062,421 (7.53%)	5,083,301 (7.56%)
LS	LS-1	69,772,862	47,954,421 (68.73%)	44,978,907 (64.46%)	2,975,514 (4.26%)	25,841,250 (37.04%)	25,972,897 (37.22%)
	LS-2	64,857,218	45,245,487 (69.76%)	42,403,122 (65.38%)	2,842,365 (4.38%)	24,431,379 (37.67%)	24,529,112 (37.82%)
	LS-3	66,596,720	46,187,953 (69.35%)	43,318,071 (65.05%)	2,869,882 (4.31%)	24,903,147 (37.39%)	25,021,386 (37.57%)

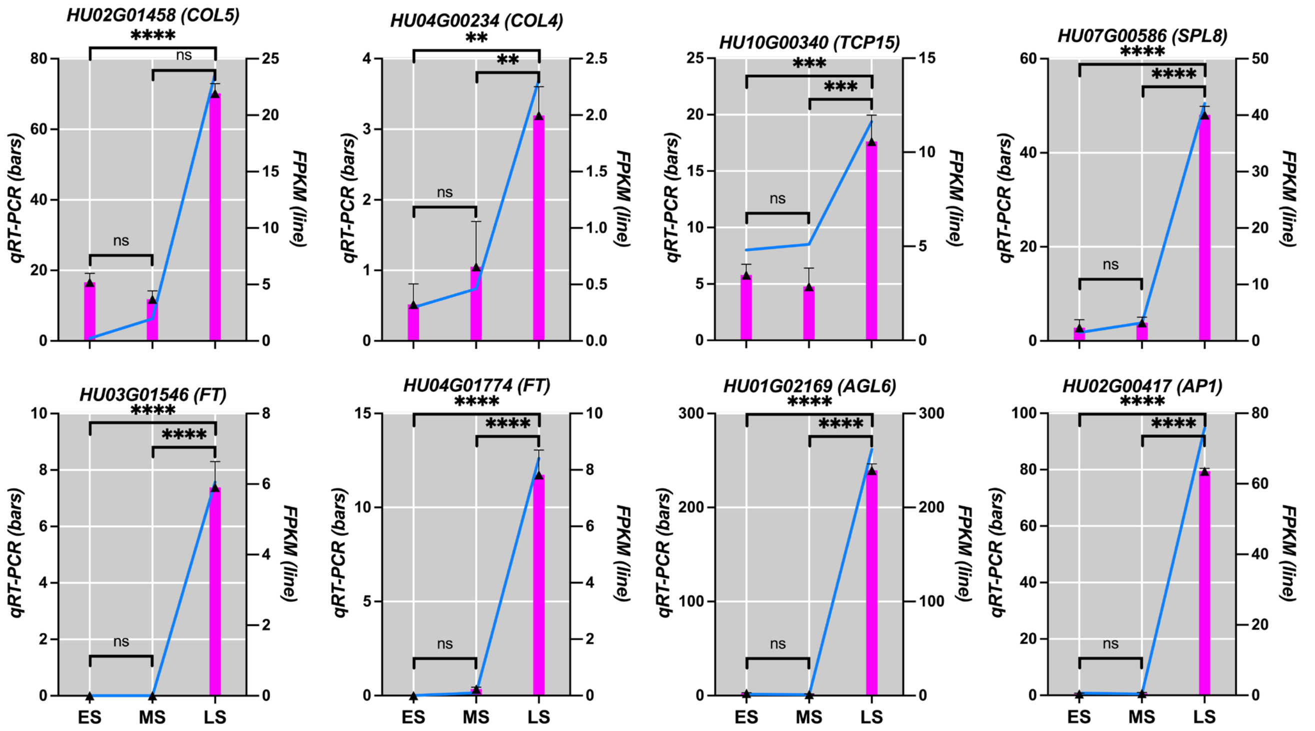

Figure S1. qRT-PCR validation of selected transcription factors and structural flowering genes. Bars indicate gene expression values detected by qRT-PCR, while the lines represent the gene expression values from transcriptome sequencing. Data are presented as mean ± SD for three replicates ($n = 3$). Significant differences were determined by t-tests using Graphad Prism 10 for macOS (Version 10.1.1) and denoted as follows: * $p < 0.05$; ** $p < 0.01$; *** $p < 0.001$; **** $p < 0.0001$; nonsignificant differences are indicated by 'ns' ($p > 0.05$).
